# Genomic, Lipidomic and Metabolomic Analysis of Cyclooxygenase-null Cells: Eicosanoid Storm, Cross Talk, and Compensation by COX-1

**DOI:** 10.1016/j.gpb.2014.09.005

**Published:** 2016-03-21

**Authors:** Abul B.M.M.K. Islam, Mandar Dave, Sonia Amin, Roderick V. Jensen, Ashok R. Amin

**Affiliations:** 1 Department of Genetic Engineering and Biotechnology, University of Dhaka, Dhaka 1000, Bangladesh; 2 Department of Rheumatology, New York University Hospital for Joint Diseases, New York, NY 10003, USA; 3 Department of Biology and Chemistry, Essex County College, Newark, NJ 07102, USA; 4 Department of Biological Sciences, College of Science, Virginia Tech Blacksburg, VA 24060, USA; 5 Department of Pathology, New York University School of Medicine, New York, NY 10016, USA; 6 RheuMatrix Inc., Blacksburg, VA 24060, USA

**Keywords:** Prostaglandins, Metabolomics, Lung fibroblasts, Genomics, Inflammation

## Abstract

The constitutively-expressed cyclooxygenase 1 (COX-1) and the inducible COX-2 are both involved in the conversion of arachidonic acid (AA) to **prostaglandins** (PGs). However, the functional roles of COX-1 at the cellular level remain unclear. We hypothesized that by comparing differential gene expression and eicosanoid metabolism in **lung fibroblasts** from wild-type (WT) mice and COX-2^-/-^ or COX-1^-/-^ mice may help address the functional roles of COX-1 in **inflammation** and other cellular functions. Compared to WT, the number of specifically-induced transcripts were altered descendingly as follows: COX-2^-/-^ > COX-1^-/-^ > WT + IL-1β. COX-1^-/-^ or COX-2^-/-^ cells shared about 50% of the induced transcripts with WT cells treated with IL-1β, respectively. An interactive “anti-inflammatory, proinflammatory, and redox-activated” signature in the protein–protein interactome map was observed in COX-2^-/-^ cells. The augmented *COX-1* mRNA (in COX-2^-/-^ cells) was associated with the upregulation of mRNAs for glutathione *S*-transferase (GST), superoxide dismutase (SOD), NAD(P)H dehydrogenase quinone 1 (NQO1), aryl hydrocarbon receptor (AhR), peroxiredoxin, phospholipase, prostacyclin synthase, and prostaglandin E synthase, resulting in a significant increase in the levels of PGE_2_, PGD_2_, leukotriene B_4_ (LTB_4_), PGF_1α_, thromboxane B_2_ (TXB_2_), and PGF_2α_. The COX-1 plays a dominant role in shifting AA toward the LTB_4_ pathway and anti-inflammatory activities. Compared to WT, the upregulated *COX-1* mRNA in COX-2^-/-^ cells generated an “eicosanoid storm”. The genomic characteristics of COX-2^-/-^ is similar to that of proinflammatory cells as observed in IL-1β induced WT cells. COX-1^-/-^ and COX-2^-/-^ cells exhibited compensation of various eicosanoids at the genomic and metabolic levels.

## Introduction

Eicosanoids are lipid mediators of inflammation [1]. Cytosolic phospholipase A_2_ (cPLA_2_) cleaves some membrane lipids to generate 20-carbon arachidonic acid (AA), which can be channeled toward eicosanoid synthesis [1]. The generation of AA by cPLA_2_ is a rate-limiting step for biosynthesis of eicosanoids, which include prostaglandins (PGs), thromboxanes (TXs), and leukotrienes (LTs). PGs and TXs, which are collectively referred as prostanoids, are generated by the two isoforms of cyclooxygenases (COX), COX-1 and COX-2, whereas LTs are generated by 5-lipoxygenase (5-LOX) [1], [2]. Although both COX enzymes generate PGs, COX-1 is constitutively expressed in most normal cells at the basal level and is considered to regulate a number of housekeeping functions such as vascular hemostasis, renal blood flow, and glomerular function [1], [2]. On the other hand, COX-2, which is inducible in nature, is mostly expressed at sites of inflammation and cancer [1], [2]. Expression of COX-2 can be triggered by cytokines, growth factors, and other proinflammatory stimuli [2]. Cell- and/or tissue-specific gene expression of COX-1 and COX-2 has been reported in different organs and tumors [3], [4], [5]. Moreover, COX-2 overexpression induces tumors in COX-2 transgenic mice, whereas the COX-2 inhibitors coxibs induce oxidative stress and reduce polyps associated with colon cancer [2], [6], [7].

Fibroblasts have a wide distribution throughout the body. Owing to their ability to secrete extracellular matrix (*e.g.*, collagen) and release proinflammatory mediators [8], fibroblasts are involved in various pathophysiological conditions such as arthritis, cancer, lung fibrosis, wound healing, and stem cell maturation [8]. Fibroblastic COX-1 and COX-2 activity [8] can be modulated by various receptors such as PGE receptors (EP receptors) and Toll-like receptors (TLRs) [1], [2], [8]. These receptors may influence eicosanoid synthesis, growth, chemotaxis, matrix, and matrix metalloprotease synthesis [1], [2]. We therefore chose to use fibroblasts over macrophages for the present study, in line with our previous investigations on the regulation of COX and PGs in peripheral blood leukocytes, chondrocytes (differentiated fibroblasts), and synovial fibroblasts in human arthritis [9], [10], [11]. We expect our systems approach (genomic, metabolomic, protein-interatomic, and proteomic) from the same cells will allow identification of common and distinct functions of the two closely related COX isozymes [12], [13] in sterile inflammation, innate immune response, and homeostasis in the cells.

The uncontrolled production or defective expression of COX-1 or COX-2 has been recognized as a health risk [14], [15]. Similarly, nonsteroidal anti-inflammatory drugs (NSAIDs) and COX-2 inhibitors (coxibs) also exhibited health risk in large-scale population studies [16], [17]. The Food and Drug Administration (FDA) of the United States has recommended that coxibs be avoided in individuals with an elevated risk of cardiovascular disease (CVD) and in patients with established CVD [16], [17], since the drugs increased the risk of ischemic CVD, heart failure, increased blood pressure, and cardiac arrhythmia [2], [16], [17]. One explanation of these adverse events is the differential accumulation of TXs, LTs, and PGs in the presence of coxibs and/or decreased level of prostacyclins [2], [6], [7], [8], [9], [10], [11] and/or “non-PG effects” of COX inhibitors [18], [19], [20]. Inhibition of COX-2 by coxibs in human arthritis not only curbs COX-2-mediated PGs but also shifts prostanoid synthesis toward the COX-1-mediated pathway [3], [10]. Unlike COX-2, the participation of COX-1 has been subverted, for its involvement in inflammation [1], [2]. For example, the anti-inflammatory effects of a highly-selective coxib were evident only when administered at doses that are inhibitory for COX-1, indicating a substantial contribution of COX-1 in inflammation [2].

IL-1β is a proinflammatory factor with a wide spectrum of metabolic, physiological, homeostatic, inflammatory, sterile inflammatory, and immune activities in diseases [21], [22], [23]. Sterile inflammation is caused by cell damage in trauma, ischemia, ischemia–reperfusion, *etc*. via endogenous ligands, such as high-mobility group protein B1(HMGB1), amyloid, S100 proteins, and heat shock proteins (HSPs) [24], [25]. It has been implicated in complex diseases, with the upregulation of IL-1 and eicosanoids as the common denominator [1], [9], [21], [22], [23].

In the present study, we report the alternation in gene expression and end products of lipid synthesis for COX-1, COX-2, and 5-LOX pathways in COX-1^-/-^ (COX-1-ablated) and COX-2^-/-^ (COX-2-ablated) cells. We demonstrate the dominant role of COX-1 at the genomic, redox, and metabolomic levels in COX-2-null cells. In addition, gene array data also agree with the metabolic activity of eicosanoids and redox changes in COX-2^-/-^ cells.

## Results

### Gene expression arrays and hierarchical clustering in mouse fibroblasts

In the present study, we utilized knockout cells to eliminate the variables previously observed when using COX-1/COX-2 inhibitors to dissect the functional roles of COX-1/COX-2, such as “non-PG effects”, specificity- and concentration-dependent outcomes [3], [12], [13], [16], [17], [18], [19], [20].

**Figure 1:**
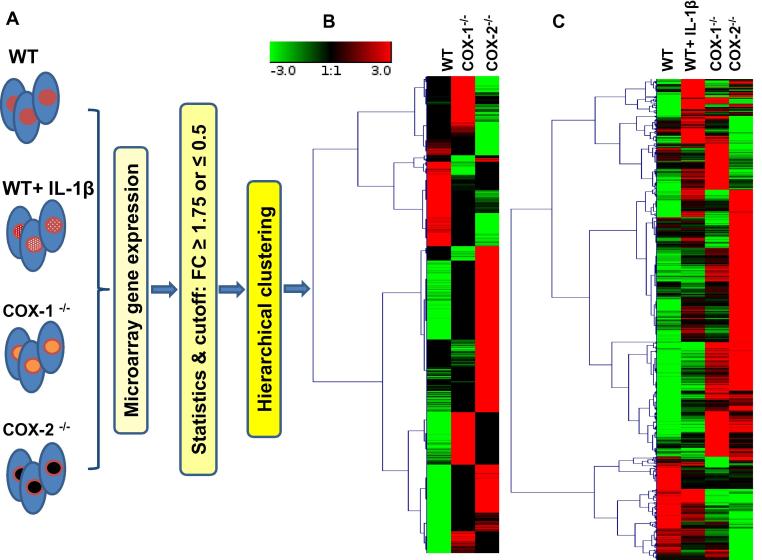
**General scheme for experimental conditions, bioinformatics analysis, and hierarchical clustering of genes** Gene expression arrays were performed on wild-type control (WT), WT + IL-1β, COX-1^-/-^, and COX-2^-/-^ fibroblasts (**A**). Hierarchical clustering was performed using median-centered gene expression among WT, COX-1^-/-^, and COX-2^-/-^ cells (**B**), as well as WT, WT + IL-1β, COX-1^-/-^, and COX-2^-/-^ cells (**C**). In the heat map, the color toward green indicates lower expression from median while red indicates higher expression from the median center. The heat map shows differentially-expressed transcripts (FC ⩽0.5 or ⩾1.75) only. FC, fold change.

Mouse fibroblast cells were obtained from WT cells (WT), COX-1^-/-^, and COX-2^-/-^ mice. In addition, WT cells stimulated with 10 ng/ml IL-1β served as a positive control to procure an “IL-1β inflammatory signature” at the genomic level. All the cells were subjected to gene expression arrays [26], [27], [28] and as shown in Figure 1**A**. We implemented bioinformatics analysis to identify specific transcripts that were upregulated by 1.75-fold or downregulated by 0.5-fold in COX-1^-/-^, COX-2^-/-^, and IL-1β stimulated WT cells, respectively, when compared to basal gene expression in WT cells. Hierarchical clustering of gene transcripts showed altered gene expression in COX-1^-/-^ and COX-2^-/-^ cells as compared to WT cells (Figure 1**B**; Figure S1 for high resolution and gene annotations). These also include *COX-1* and genes involved in eicosanoid synthesis, inflammation, homeostasis, and cell cycle in COX-2^-/-^ cells.

As a result, we found expression of 223 transcripts was up- or downregulated in IL-1β-induced WT cells (WT + IL-1β), representing the “IL-1β inflammatory signature” (Figure 1**C**, Figure S2, and Table S1). About 50% of these 223 transcripts were also modulated as the IL-1β inflammatory signature in COX-1 or COX-2 ablated cells (Figure 1C, S2 and Table S1). Expression of some highly or lowly modulated transcripts by IL-1β [21], [22], [23], [24], [25], such as acute phase protein serum amyloid A3 (SAA3) and IL33 was further plotted (Figure S3). As expected, IL-1β induced the expression of *COX-2* but not *COX-1*, comparing WT *vs.* WT *+* IL-1β cells [21], [22], [23], [24], [25].

### Gene expression arrays of eicosanoid metabolism

**Figure 2:**
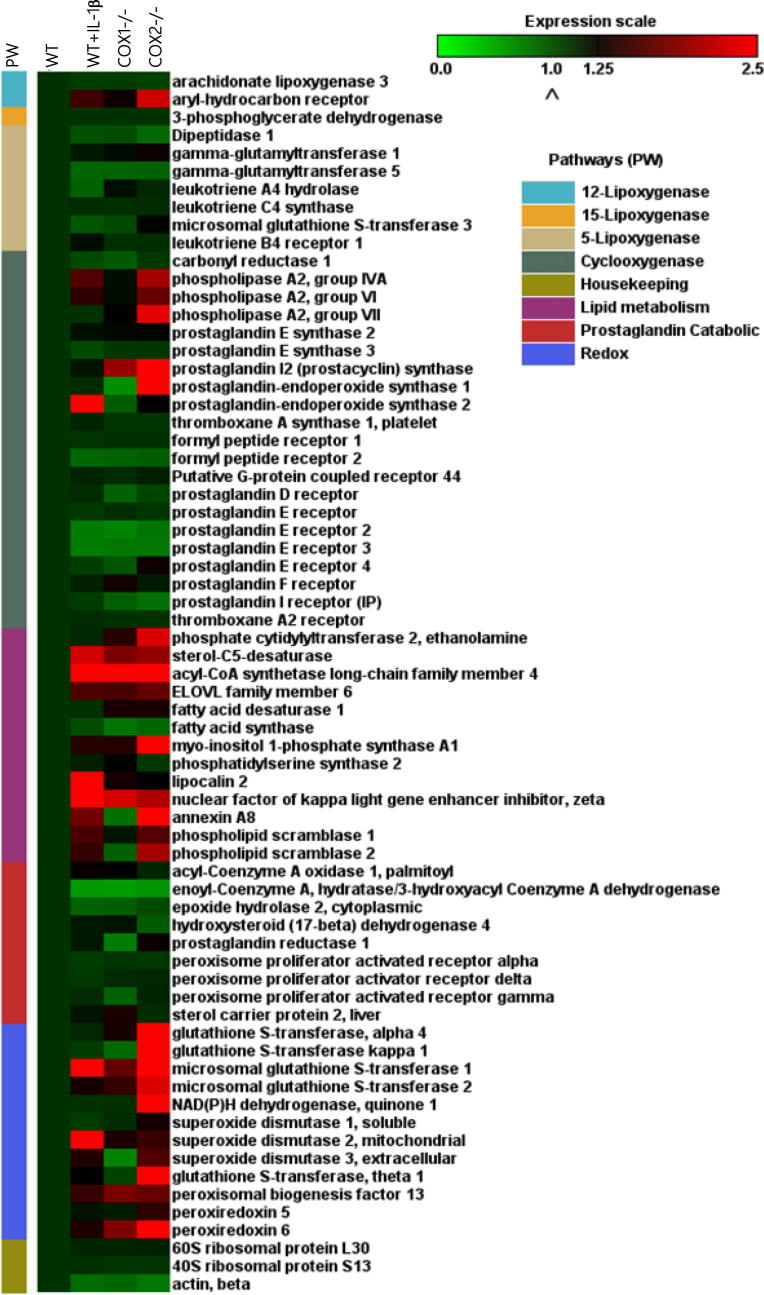
**Gene expression of eicosanoid metabolism in COX-1^-/-^, COX-2^-/-^, WT, and WT + IL-1β cells** The differentially-expressed transcripts were clustered into different pathways. Gene expression is represented as fold change in relation to WT cells from low to high in color gradient (from green to red). Red indicates a higher expression, while green indicates a lower expression. The average baseline fold change of all the three housekeeping transcripts was 1.0 ± 0.3.

To understand the role of COX-1, we focused on the alterations in gene expression in COX-2^-/-^ cells. Sixty-five transcripts were identified to be involved in eicosanoid, lipid, and redox metabolism pathways. These transcripts were grouped into different eicosanoid pathways as shown in the heat map of differentially-expressed transcripts from WT, WT + IL-1β, COX-1^-/-^, and COX-2^-/-^ cells (Figure 2). More than 25% of these 65 transcripts that were upregulated in IL-1β stimulated WT cells were also spontaneously augmented in COX-2^-/-^ cells.

The genes of PLA_2_-group [29], including *Pla_2_g4*, *Pla_2_g6*, and *Pla_2_g7*, were highly expressed in COX-2^-/-^ followed by COX-1^-/-^, IL-1β + WT, and WT cells (Figure 2 and Table S2). Compared to WT and COX-1^-/-^ cells, increased expression of genes encoding PGE synthase (*Ptges*) and PGI_2_ (as called prostacyclin) synthase (*Ptgis*) was detected in COX-2^-/-^ cells. Glutathione *S*-transferases (GSTs) are key enzymes in the synthesis of various LTs [30], [31]. There was an increase in genes encoding various GST isoforms, including GST theta 1 (*Gstt1*), GST alpha 4 (*Gsta4*), GST kappa 1 (*Gstk1*), and microsomal GST 1 and 2 (*Mgst1*, *Mgst2*), in COX-2^-/-^ cells as compared to WT, WT + IL-1β, and COX-1^-/-^ cells. Similarly, increased expression was also revealed for genes encoding superoxide dismutase (*Sod*), peroxiredoxin (*Prdx*), and proteins involved in the phospholipid translocation, such as scramblase 2 (*Plscr2*) [30], [31], [32], [33].

### Network analysis

**Figure 3:**
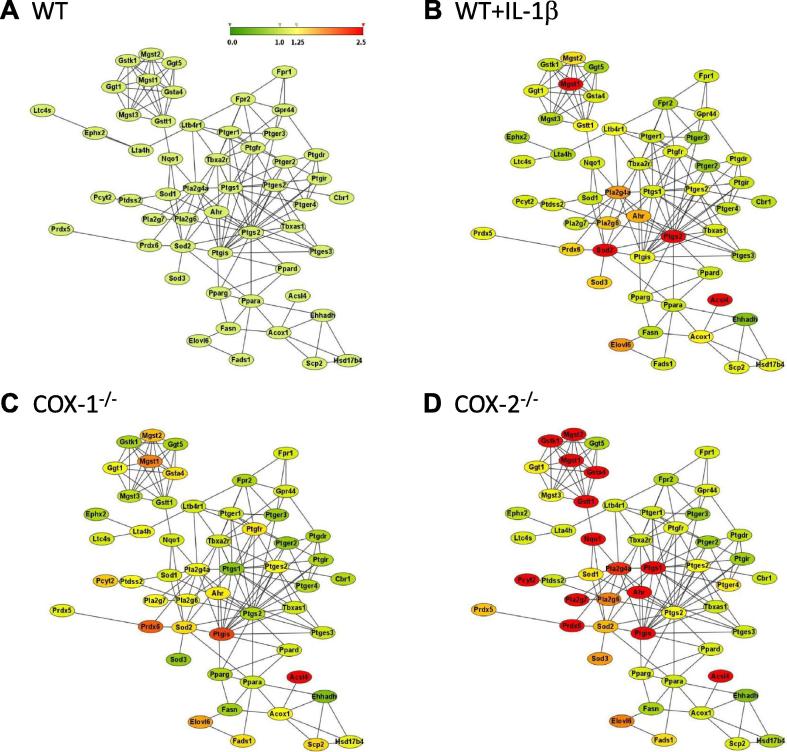
**Protein–protein interactions in eicosanoid metabolism** Protein–protein interactome map of the selected group of eicosanoid metabolism genes are presented in a network model in WT (**A**), WT + IL-1β (**B**), COX-1^-/-^ (**C**), and COX-2^-/-^ (**D**) cells. Genes/proteins are represented as nodes and interactions are represented as edges. Gene expression is represented as fold change in relation to WT cells from low to high in color gradient (from green to red; fold change = 0–2.5). Fold change is set as 1 for WT. The highest intensity red color in this scale indicates that fold change ⩾2.5-fold that of basal levels.

The Database Search Tool for the Retrieval of Interacting Genes/Proteins (STRING) contains information on about 5.2 million proteins in 1133 species from experimental data, computational prediction methods, and public text collections. Data are weighted and integrated with a confidence score calculated for all protein interactions. STRING allows merging of gene expression arrays and metabolic activity at the level of protein–protein interactome [13], [34]. Using the edge information present in the STRING database, protein–protein interaction map (Figure 3) was derived from the gene expression data in this study. In WT cells, there exist interactions of different proteins in the eicosanoid pathways (Figure 3A), representing the background signal of WT cells. The map shows the interactions among the functional genes/proteins associated with 5-, 12-, and 15-LOXs, COX-1 and COX-2, and lipid metabolism. Figure 3 showed an increase in gene/protein activities in the following order of magnitude: COX-2^-/-^ > COX-1^-/-^ = IL-1β + WT > WT cells. The differences in redox-related protein were more pronounced in COX-2^-/-^ cells (Figure 3D). For example, expression of NAD(P)H dehydrogenase quinone 1 (NQO1) and its receptor aryl hydrocarbon receptor (AhR) was increased in COX-2^-/-^ as compared to WT, COX-1^-/-^, and IL-1β-stimulated cells. *NQO1* is induced during cellular stress, detoxification, tumors, tardive dyskinesia, and hematotoxicity [35], [36]. AhR has been shown to regulate xenobiotic-metabolizing enzymes such as cytochrome P450, which is involved in eicosanoid metabolism [36]. Taken together, these interaction maps showed that unlike WT and COX-1^-/-^ cells, COX-2^-/-^ cells exhibited a distinct protein–protein interaction signature.

### Lipidomic studies and strategy

#### Accumulation of PGE_2_ and PLA_2_ in COX-1^-/-^ and COX-2^-/-^ cells

**Figure 4:**
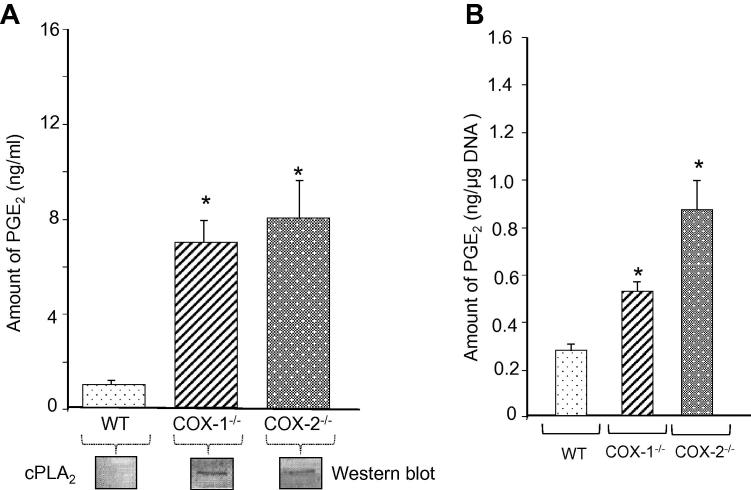
**Spontaneous production of PGE_2_** The spontaneous release of PGE_2_ in cell supernatant was examined by radioimmunoassay (RIA). The amount of PGE_2_ in COX-1^-/-^ or COX-2^-/-^ was presented in relation to WT cells (**A**) and after normalized with cellular DNA (**B**). The protein levels of cPLA2 in different cell samples were detected using Western blotting. Data are expressed as mean ± SEM (*n* = 3 for each experiment). Student’s *t-*test was performed for statistical analysis and significant differences are labeled with ^∗^(*P* < 0.01) when compared to WT cells.

We integrated the omics analysis as shown in Figure S4 [37]. We next selected stable end-metabolites (from WT and COX mutants), which can be quantitatively assayed and may also represent pathways involved in eicosanoid synthesis. The expression levels of genes encoding isoforms of PLA_2_ of the groups VII (*Pla_2_g7*), IVA (*Pla_2_g4)*, and VI (*Pla_2_g6*) were spontaneously upregulated in the following order: COX-2^-/-^ > COX-1^-/-^ = IL-1β + WT cells (Figure 2 and Table S2). Basal levels cPLA_2_ were not detected by western blot analysis in unstimulated fibroblasts in this and other studies [3], [29]. Nevertheless, COX-1^-/-^ and COX-2^-/-^ cells (and WT + IL-1β cells [3], [38]) showed upregulation of cPLA_2_ as determined by Western blot analysis (Figure 4). These data not only confirmed previous observations [29] with respect to the functional role of cPLA_2_, but also identified new classes of PLA_2_: the lipoprotein-associated phospholipase A_2_ (Lp-PLA_2_) encoded by *Pla_2_g7*, which is associated with eicosanoid metabolism in these mutant fibroblasts.

Consistent with the upregulated expression of cPLA_2_ in COX-ablated cells, the levels of PGE_2_ were significantly increased (3–4-folds) in COX-1^-/-^ and COX-2^-/-^ cells, although it is present at a basal level in WT cells (Figure 4A). Similar observations were made after normalizing the amount of PGE_2_ with the cellular DNA content (Figure 4B). We obtained the similar results as those reported by Ballou et al. [3]. The increased levels of PGE_2_ was accompanied by increases in the expression of *Ptgs1*, *Ptgs2*, *Acsl4*, *Elovl6*, *Fads1*, *Plscr1*, *Plscr2*, *Ptgis*, and *Ptges*, which are involved in lipid metabolism (Figure 2 and Table S2). These results suggest an increase in the synthesis and compensation of the dominant PG (*e.g.*, PGE_2_) with other enzymes in lipid metabolism by COX-1 and COX-2 pathways in the absence of either COX isoforms.

#### Regulation of PGE_2_ in COX-1- and COX-2-ablated cells

**Figure 5:**
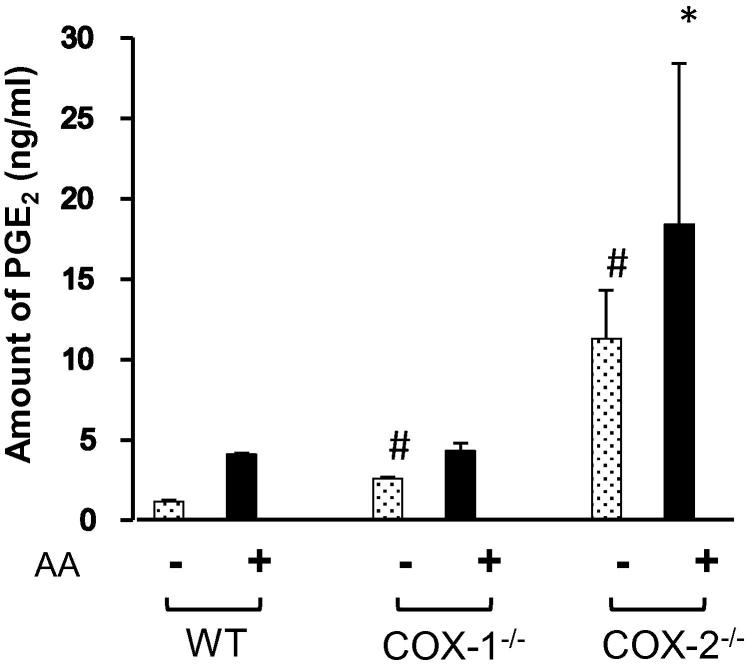
**Regulation of PGE_2_ in the presence or absence of AA** Amount of spontaneously-released PGE_2_ was measured using RIA in the supernatant of WT, COX-1^-/-^, and COX-2^-/-^ cells with 0.5 μM of AA or without AA treatment for 15 min. Data are expressed as mean ± SEM (*n* = 3 for each experiment). Student’s *t-*test was performed for statistical analysis. Significant differences are labeled with ^#^(*P* < 0.05) when compared to WT cells. The significant difference between AA-treated WT and COX-ablated cells is labeled with ^∗^(*P* < 0.05). AA, arachidonic acid.

We then examined if the expression of COX-1 and COX-2 was sensitive to inhibitors (indomethacin) and activators (IL-1β and/or AA) of COX-1 and COX-2 in WT, COX-1^-/-^, and COX-2^-/-^ cells (Table 1). We found that indomethacin significantly inhibited PGE_2_ accumulation in COX-1 or COX-2-ablated cells, confirming that both enzymes were independently involved in the generation of PGE_2_ [1], [2]. On the other hand, we also tested the effects of the endogenous activator AA (Figure 5) and found that AA significantly increased the levels of PGE_2_ in WT and COX-1^-/-^ cells, but not in COX-2^-/-^ cells. These experiments show the importance of endogenous AA as a rate limiting step for regulation of COX-1 and COX-2 pathways [1], [2], [3], [39].

#### Analysis of prostanoids in COX-1^-/-^ and COX-2^-/-^ cells

**Figure 6:**
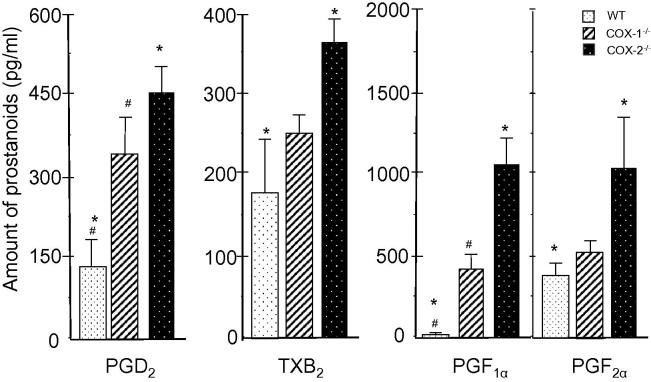
**Differential synthesis of prostanoids in WT, COX-1^-/-^, and COX-2^-/-^ cells** Amount of PGD_2_, TXB_2_, PGF_1α_, and PGF_2α_ in cell supernatant was examined by ELISA at 24 h of incubation. Data are expressed as mean ± SEM (*n* = 3 for each experiment). Significance levels were calculated using Student’s *t-*test. Significant differences in the amount of prostanoids between COX-ablated and WT cells are indicated with ^∗^ for COX-2^-/-^ cells and ^#^ for COX-1^-/-^ cells, respectively (*P* < 0.05)*.*

PGE_2_ is one of the dominant prostanoids in COX-ablated fibroblasts as observed in other cell types [1], [2]. We examined the production of PGD_2_, TXB_2_, 6-keto-PGF_1α_, and PGF_2α_, which has not been examined previously in these COX mutants (Figure 6). The amount of PGD_2_ was significantly increased in the COX-1^-/-^ and COX-2^-/-^ cells as compared to that in WT cells. Compared to WT cells (around 170–180 pg/ml), the amount of TXB_2_, a stable and an inactive metabolite of TXA_2_, was significantly increased in COX-2^-/-^ but not COX-1^-/-^ cells. Different from PGD_2_ and TXB_2_, PGF_1α_ was identified in low concentrations in WT cells, but increased significantly in both COX-1^-/-^ and COX-2^-/-^ cells. On the other hand, PGF_2α_ levels were relatively high in WT cells but significantly increased in COX-2^-/-^ cells, while only marginal increase was detected in COX-1^-/-^ cells. In addition, there was a 1–5-fold increase in several prostanoids (like PGE_2_) in the presence of exogenous AA (data not shown). In summary, as compared to WT cells, the levels of TXB_2_ and PGF_2α_ were significantly higher in COX-2^-/-^ cells but not COX-1^-/-^ cells. There was a compensation of basal levels of PGE_2_, PGD_2_, and PGF_1α_ in COX-1^-/-^ and COX-2^-/-^ cells due to increased activity of cPLA_2_ and high levels of common substrate: AA in both mutants. This leads to an imbalance in the basal levels of prostanoids in the mutants (by the COX-1 and COX-2 pathways) as compared to WT cells.

#### Cross-talk between COX and 5-LOX pathways in COX-2^-/-^ cells

**Figure 7:**
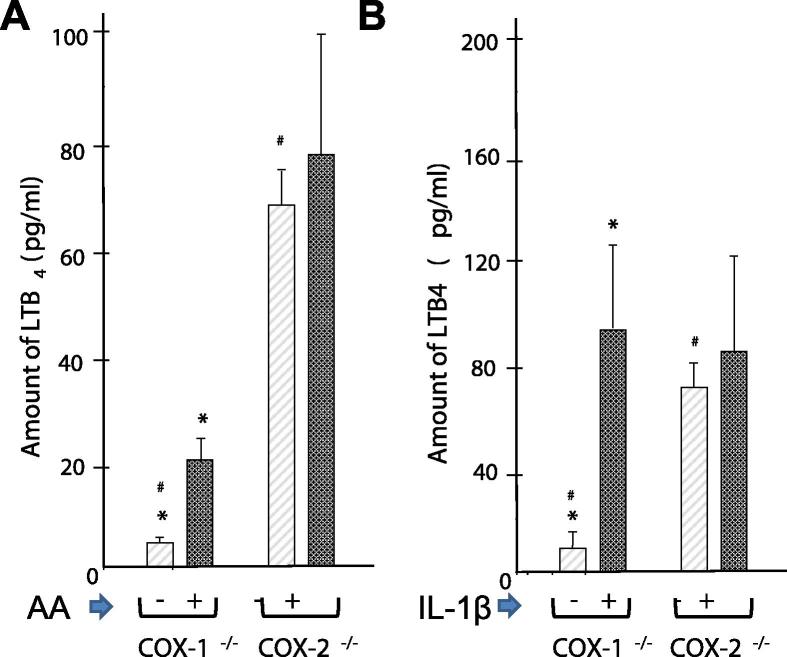
**Effect of AA and IL-1β on the level of LTB_4_ in COX-1^-/-^ and COX-2^-/-^ cells** The amount of spontaneous release of LTB_4_ in the COX-1^-/-^ and COX-2^-/-^ cells in the presence or absence of AA (**A**) or IL-1β (**B**) was evaluated by ELISA. Significant alteration in the amount of LTB_4_ (*P* *<* 0.05) between COX-1^-/-^ and COX-2^-/-^ cells is indicated with ^#^, while difference between the treated and untreated samples in the same cell groups is indicated with ^∗^. All data are expressed as mean ± SEM (*n* = 3). Significance levels were calculated using Student’s *t-*test.

The shift from PGE_2_ to LTB_4_ in cytokine-activated cells in the presence of coxibs has been reported in human arthritis-affected tissues and cells [9], [40]. In the present study, we compared the contributions of COX-1 and COX-2 in the 5-LOX pathways with and without any stimulation (Figure 7). COX-2^-/-^ and COX-1^-/-^ cells showed a spontaneous release of LTB_4_, although it was much higher in COX-2^-/-^ cells. In addition, extra AA or IL-1β significantly increased the levels of LTB_4_ accumulation in COX-1^-/-^ cells, which is not the case for COX-2^-/-^ cells. In summary, we demonstrated the differential and preferential contribution of LTB4 synthesis by the COX-1 pathway over the COX-2 pathway.

## Discussion

The present study describes the common and dissimilar functional interactions of the COX isozymes at the cellular level. Previous pharmacogenomics analysis showed inhibition of PGE_2_ by coxib in human arthritis-affected blood cells and cartilage, resulted in altered gene expression [9], [10], [11]. Coxibs or statins with different chemical structures and similar pharmacological targets, elicited distinct side effects, changes in protein profile and gene expression [1], [2], [16], [17], [18], [19], [41], [42], [43]. It should be noted that altered gene expression incited by coxibs may not necessarily account for the inhibition of COXs and PGs, since some effects of coxib treatment cannot be reversed by the addition of exogenous prostanoids [18], [19], [20], [42], [43]. Furthermore, the differential physiological actions of coxibs for curbing pain and inhibiting colon cancer are concentration dependent [1], [2].

### Role of COX-1 and COX-2 in inflammation and inflammation resolution

IL-1β and other members of the growing superfamily of IL-1 are involved in sterile inflammation under various pathophysiological conditions [21], [22], [23], [24], [25]. Approximately 50% of transcripts modulated in COX-1- or COX-2-ablated cells were also observed in the “IL-1β inflammatory signature”. Since inflammasome and COX-2 pathways are induced by IL-1β, the intricate roles of COX-2 during sterile inflammation and innate immunity were not surprising [23], [24]. However, the inflammatory response exhibited by the upregulated COX-1 expression (in the absence of COX-2 activity) was certainly perplexing. These results further support the previously overlooked role of COX-1 within and beyond eicosanoid metabolism and inflammation. Others and we have shown that low levels of PGE_2_ exhibit proinflammatory activity by activating NFκB pathway [44]. However, prolonged activation of COX by IL-1β and high levels of PGE_2_ foster inflammation resolution by inhibiting the NFκB pathway [45], [46]. Prolonged and a constitutive upregulation of PGE_2_ (in COX-ablated cells and IL-1β-stimulated WT) triggered the upregulation (by 2–3-fold) of a gene (*Nfkbiz*), which inhibits NFκB functions [44].

COX-1 or COX-2 in collaboration with other mediators may participate in inflammation, inflammation resolution, and cancer [1], [2], [45], [46]. The multifunctional transcription factor AhR regulates many genes including *COX-2*, but not *COX-1* [36], [47], [48], [49], [50], [51], [52]. *AhR*^-/-^ mice have been reported to develop heightened inflammatory responses with decreased induction of COX-2, lipid peroxidation, and oxidative stress [52]. AhR agonist leflunomide, which is used for the treatment of rheumatoid arthritis (RA), exhibits anti-inflammatory activity [51]. However, another agonist of AhR, TCDD, induces *COX-2* (but not *COX-1*) transcription in cancer due to the presence of the AhR binding site (xenobiotic response elements, XRE) in the *COX-2* promoter region [36], [47]. The upregulation of AhR was only observed in COX-2^-/-^ but not in COX-1^-/-^ cells. The COX-2^-/-^ cells (with amplified COX-1 activity) also exhibited increased oxidative stress with elevated levels of NQO1, GSTs, PRDX2, and SODs. The distended levels of AhR in COX-2^-/-^ cells may participate in not only detoxification [35], [36], but also anti-inflammatory activity [51]. These preliminary observations require further experimental studies to better understand the functional relationships between AhR and COX-1.

### Role of PLA***_2_*** isoforms and COX-1 in inflammation

In the current studies, the differential expression of various PLA_2_ isoforms in COX-1^-/-^ and COX-2^-/-^ cells were induced by the exclusive COX-2 or COX-1 pathways, respectively. The current literature strongly implicates PLA_2_G4 as the primary enzyme in polyunsaturated fatty acid release for eicosanoid biosynthesis [12], [29]. *Pla2g4*^-/-^ mice were not able to produce eicosanoids [29], [37]. The upregulated *Pla2g4* in IL-1β-induced WT, COX-1^-/-^, and COX-2^-/-^ cells accounted for the increased eicosanoid synthesis [3], [29], [37]. Transfection of *Pla2g6* results in upregulation of PGE_2_ and PGF_2α_ [29], [37]. Indeed, unlike in COX-1^-/-^ cells, an increase in *Pla2g6* was associated with increased levels of PGF_2α._ in COX-2^-/-^ cells in the present study. Lp-PLA_2_ (*Pla2g7*) was upregulated in COX-2^-/-^ cells and plaque forming inflammatory cells in atherosclerosis [29], [37]. The differential expression of isoforms of PLA_2_ supports the distinct role of COX-1 in mounting an inflammatory response in the absence of COX-2.

Our studies suggest that COX-1 upregulates gene expression and metabolic activity, which results in a significant increase in the synthesis of prostanoids and redox-related activity in COX-2^-/-^ cells. It should be noted that COX-2^-/-^ fibroblasts showed a nearly fourfold increase in *COX-1* gene expression as compared to WT cells. Although COX-1 is regarded as a “constitutive enzyme” [1], [2], [3], *COX-1* gene expression is upregulated in the colon and ovarian cancers [53], [54]. Estradiol also stimulates gene expression of PLA_2_ and COX-1 in endothelial cells [55]. Von Moltke et al. studied the role of COX-1 during systematic inflammation induced by flagellin in mice [56]. They showed that COX-1-derived products drive the initial phase of the inflammatory process, whereas COX-2 upregulation followed a few hours later [56]. These observations highlight the new role of COX-1-generated eicosanoids and physiological consequences *in vivo* and also support the hypothesis that the basal activity of COX-1, when placed in a pivotal position, can mount an inflammatory response with an “eicosanoid storm” to compensate for the multifunctional eicosanoids, lipids, and redox-related mediators.

### Regulation of metabolites by COX-1, COX-2, and LOX

COX-1 and COX-2 differ in metabolic functions. Unlike COX-1, COX-2 can metabolize dihomo-γ-linolenic and eicosapentaenoic acid in addition to AA [37]. Another noteworthy difference between COX-1^-/-^ and COX-2^-/-^ fibroblasts is their ability to synthesize different quantities and forms of PGs, TXs, LTs, and lipids. Changes in levels of eicosanoids may regulate each other. For example, an increased production of PGD_2_ in human arthritis-affected tissues increases the accumulation of PGE_2_, PGF_1α_, PGF_2α_, and TXB_2_ but inhibits LTB_4_ [57]. The expression of COX-1 can have far-reaching effects on lipid metabolism. For example, Ma et al. reported significant changes in the levels of phosphatidylserine, triacylglycerol, and cholesterol, which alter the cholesterol-to-phospholipid ratio in COX-2^-/-^ mice and may impart resistance to neuroinflammation [58]. Thus increasing the activity of COX-1 (in COX-2^-/-^ cells) not only regulates eicosanoid synthesis but also augments lipid metabolism as described in Figure 2. An increased activity of COX-1 increased the levels of LTB_4_ in COX-2^-/-^ cells that were comparable to those induced by IL-1β- or AA-induced COX-1^-/-^ cells. Similar effects were observed in human osteoarthritis-affected cartilage that spontaneously released PGE_2_ and LTB_4_ in *ex vivo* models [9]. The proinflammatory activity of LTB_4_ is well documented in fibroblasts. Expression of TNFα and IL-1β is increased by LTB_4_ but inhibited by LTB_4_ inhibitors, such as MK886 and bestatin [1], [2].

### Increase in redox molecules in COX-ablated cells

The increase in GSTs in COX-2^-/-^ cells was higher than that in COX-1^-/-^ cells, followed by that in WT cells. GSTs contribute to the detoxification of xenobiotics and endogenous peroxided lipids, and biosynthesis of LTs [30], [31], [32]. Peroxiredoxins function as antioxidant enzymes that also control inflammation-induced peroxide levels by removing H_2_O_2_ [59]. Mice lacking peroxiredoxin develop severe hemolytic anemia and hematopoietic cancers, and have shortened lifespan [59]. Microsomal PGE synthase-1 (mPGES-1) requires glutathione as a co-factor [37]. One of the most notable observations is the increase in gene expression of *NQO1* and *AHR*. AhR-mediated *NQO1* gene expression is increased by a variety of antioxidants, tumor promoters, and H_2_O_2_ [35], [36]. COX-2^-/-^ cells showed an increase in the pentose phosphate pathway (HMP shunt, Amin AR unpublished data). The HMP shunt is a metabolic redox sensor and modulates gene expression during an anti-oxidant response [60]. These types of cellular events that lead to oxidative stress and “Warburg effect” [60] are interlinked with the regulation of COXs, NFκB activation during inflammation induces intracellular ROS [46], [47], [60]. COX-1 peroxidase activity has been reported to serve as an intracellular signal leading to NFκB activation [47], [60]. The possibility that the products of COX-1 and COX-2 may also be sensors and/or inducers for such imbalanced redox activity is compelling.

**Figure 8:**
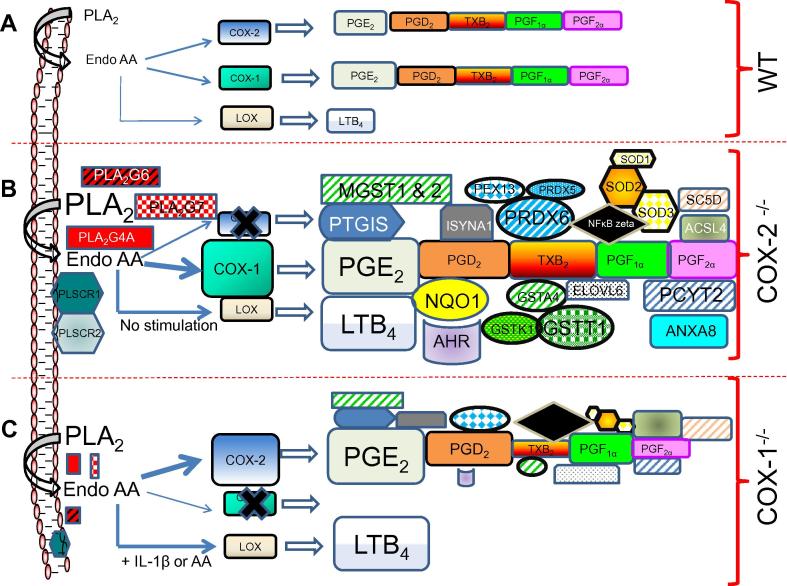
**Differential regulation of COX-1 and COX-2- in fibroblasts** As compared to the basal levels of WT (**A**), COX-2^-/-^ cells (**B**) showed an increased expression of COX-1 and various isoforms of PLA_2_, leading to upregulated PGs and LTs and compensation of the COX-2 pathway. COX-1-pathway preferentially channeled AA to PGs and LTB4 without any stimulation. COX-2^-/-^ cells also exhibited an increased gene expression associated with detoxification, anti-inflammatory and proinflammatory activities with an increased redox activity. The basal level of COX-2 in COX-1^-/-^ cells (**C**) showed compensation of PGE_2_ but not TXB_2_ and PGF_2α_. cells. LTB4 increased only in the presence of AA or IL-1β. In summary, COX-1 can not only step up to some of the functions of COX-2, but also exhibit regulation of different eicosanoids, redox reactions detoxification of free radicals, pro- and anti-inflammatory activity. Different symbol sizes represent gene and/or protein expression. Abbreviations are listed in Tables S1 and S2.

The current understanding of COX-1 in inflammation and homeostasis is constantly evolving. These studies advocate that COX-1 may participate in regulating sterile inflammation, which is more latent and subtle. There are significant collaborations and functional redundancies between COX-1 and COX-2. For example, the essential and collaborative role of both COX-1 and COX-2 has been observed in ear inflammation and arthritis models of mice [1], [61]. In addition, in the K/BxN serum–transfer model of arthritis, COX-1-derived PGs, in particular PG1_2_, contributed remarkably to initiating and prolonging the pathology [1]. Similarly, both COX isoforms contributed to PG production in the carrageenan model of inflammation, depending on the type of stimulus and tissue-specific expression of COXs [1]. The coordination and collaboration of the COXs remain well balanced, especially *in vivo*, in an inflammatory process, but may depend upon the availability of the COX isoform, time course, and intensity of the inflammation. It is apparent that if either COX isoform is functionally unavailable, the other becomes pivotal and attempts to compensate the events with a different intensity and/or tissue specificity [9], as observed in COX-1 and COX-2 knockout mice and fibroblasts [3], [4], [5], [6], [7], [8], [9], [10], [14], [15], [39]. Figure 8 summarizes the changes observed in WT, COX-2^-/-^, COX-1^-/-^ cells based on genomics and metabolomics studies described in this study These investigations paint a broader picture of eicosanoid synthesis and signaling, gene expression, and lipid metabolomics, which can identify and connect unforeseen functions of COX-1 and COX-2.

## Conclusion

This study describes the functional role of COX-1 and COX-2 at the cellular, metabolic, and genomic levels. Dysregulation of COX-1 or COX-2 results in a burst of gene expression within and beyond eicosanoid and redox metabolism in fibroblasts. The changes in basal gene expression of upregulated COX-1 in COX-2^-/-^ cells exhibited a mixture of pro- and anti-inflammatory response, as well as an activated redox signature at the genomic level. The basal levels of COX-1 > COX-2 are involved in channeling LTs from COX to 5-LOX pathway. There is compensation of eicosanoids and redox metabolism by COX-1 and COX-2 and *vice versa*. This systems approach allows the integration of genomic, bioinformatic, and lipidomic datasets. It opens new areas of biomarkers and COX regulation beyond eicosanoid metabolism, which would help to develop safer next generation of coxibs by taking into consideration the board impact of *COX* genes on other metabolic pathways.

## Materials and methods

### Reagents

Cell culture media and fetal calf serum (FCS) were obtained from Gibco BRL (Invitrogen, Carlsbad, CA). ELISA kits for the detection of PGs (PGD_2_, PGF_1α_, and PGF_2α_), TXs (TXA_2_), and LTs (LTB_4_) and AA were purchased from Chemicon International (Temecula, CA). ELISA was used to measure the levels of prostanoids and LTB_4_ [8], [9]. PGE_2_ was estimated by radioimmunoassay (RIA) as previously reported [9], [62], [63]. IL-1β was purchased from Pepto Tech (Princeton, NJ).

### Isolation and culture of COX-deficient mouse cells

Lung fibroblast cells were collected from WT C57BL/6J (B6), as well as COX-1 and COX-2 deficient mice [3], [4], [5]. The COX-1^-/-^ and COX-2^-/-^ cell lines were immortalized and cultivated from fibroblasts as previously reported [3], [4], [5], [62]. Briefly lung tissues were dissected into small pieces and grown in MEM medium [3], [4], [5], [62], with 10% FCS in a humidified incubator with 5% CO_2_. After 3 weeks of culture, only fibroblasts continued to grow. The cells were maintained in DMEM containing high glucose and supplemented with Pen/Strep (100,000 U/l penicillin G and 100 mg/l streptomycin sulfate), nonessential amino acids (0.1 mM), fungizone (1 mg/l amphotericin B), glutamine (292 mg/l), ascorbic acid (50 mg/l), and 10% FCS. The cells were incubated with 0.5 μM of AA or 10 ng/ml of IL-1β as endogenous or receptor mediator inducer of eicosanoids.

### Labeling and hybridization of microarray gene chips

Total RNA was isolated and labeled with the ENZO BioArray High-yield RNA transcript labeling kit (Affymetrix, Santa Clara, CA) and then hybridized using U74Av2 Murine Genome Array (Affymetrix) as described [26]. Genes representing 12,492 transcripts from the A chip and those annotated till 2012 were identified as present calls as per the manufacturers’ recommendations.

### Normalization of the microarray data and bioinformatics analysis

The Affymetrix microarray image files for each sample were imported into the Affymetrix Expression Console Software package (www.affymetrix.com) to calculate the robust multi-array analysis-normalized gene expression signals for 12,488 mouse gene probe sets on the U74Av2 arrays. The binary logarithm signal values were then exported into a MS Excel spreadsheet for data analysis. The log transformed expression values ranged from 6.2 to 15.4. Since the median standard deviation of the logged signal values across all probe sets for the replicate samples was small (<0.03), the fold change (FC) of the gene expression was computed by simply averaging the logged signal values for the replicate samples for comparison with the logged expression values for a single WT control sample. Our functional genomics analysis focused primarily on genes with FC ⩾1.75-fold (upregulation) and ⩽0.5-fold (downregulation), in relation to WT [26], [27], [28]. The normalization was verified using four housekeeping genes encoding glyceraldehyde-3-phosphate dehydrogenase (GAPDH) and β actin, as well as ribosomal proteins L30 (RPL30) and S13 (RPS13), which showed similar expression in all samples [64]. The heatmaps were constructed using Gitools [65] with gene expression in the WT taken as the baseline for each transcript.

### Hierarchical clustering

The hierarchical clustering of median expression value for every gene was calculated and subtracted from the absolute expression of each gene in each aforementioned experimental condition (median centering of genes). Using the tool “Genesis” and the median-centered gene expression matrix, the hierarchical cluster of genes (Pearson’s correlation distance and average linkage) was determined and the annotated data were presented as heatmaps.

### Interaction network

Interactions among proteins encoded by the selected group of eicosanoid metabolism genes were presented in a network model. Edge information among the nodes was extracted using the online tool STRING (v9.1) [66] and the interaction network was visualized using Cytoscape (v 3.1) [67].

### Eicosanoids detection in WT, COX-1^-/-^, and COX-2^-/-^ cell supernatants

Supernatants of WT, COX-1^-/-^ and COX-2^-/-^ cells were analyzed using ELISA or RIA [8], [9] to quantitate eicosanoids. The cells were seeded in triplicates unless otherwise specified and cultured for 24 h.

### Effects of AA on eicosanoid synthesis in COX-ablated cells

Cells were seeded in triplicate at a concentration of 10,000 cells/cm^2^ in 6- or 24-well plates. After treatment with 0.5 μM AA for 15 min, the cells were washed and replenished with fresh medium. The levels of eicosanoids were monitored since then and the experiments were terminated between 18 and 24 h before the fibroblasts start the exponential growth. Release of eicosanoids into the medium was subsequently estimated using ELISA or RIA as previously reported [8], [9], [63].

### Western blot analysis

The WT, COX-1^-/-^, and COX-2^-/-^ cells were cultured for 18 h, and cell extracts were prepared using a total protein extraction reagent (Pierce, Rockford, IL) as per manufacturer’s instructions and other studies [3], [62], [63]. Total protein (30 μg per sample) was separated by 10% SDS–PAGE and transferred onto a nitrocellulose membrane (Schleicher & Schuell, Keene, NH). After blocking with 3% BSA for 2 h, the blots were incubated with rabbit anti-cytosolic-PLA_2_ antibody (1:500, Santa Cruz Biotechnology, Santa Cruz, CA). Bound antibody was detected by anti-rabbit IgG-conjugated horseradish peroxidase (1:5000, Santa Cruz Biotechnology, Santa Cruz, CA) and developed using the enhanced chemiluminescence ECL plus (Amersham, Arlington Heights, IL).

### Statistical analysis

The statistical analyses were performed using GraphPad Software (v1.14) (San Diego, CA). Student’s *t-*test was used to analyze the data unless otherwise specified. Data from each experiment are represented as mean ± standard deviation. Differences between the mean values of the control and experimental groups are considered significant with *P* *<* 0.05.

## Authors’ contributions

ARA conceived the project and designed the experiments. ABI and RVJ were involved in interpretation of gene expression data and bioinformatics. SA annotated data and prepared the figures. MD performed eicosanoid assays. All authors participated in the preparation of the manuscript, read and approved the final manuscript.

## Competing interests

The project was partially supported by a Translational Research and Target Discovery Contract in collaboration with Yamanuchi Pharmaceuticals (Astellas).

## Figures and Tables

**Table 1:** Effect of indomethacin on PGE_2_ production in COX-1- and COX-2-ablated cells

**Condition**	**PGE_2_ at 24** **h (ng/ml)**	**PGE_2_ at 48** **h (ng/ml)**
*COX-1 ^-/-^ cells*		
Control (basal COX-2 activity)	6.8 ± 1.3	5.3 ± 2.6
Indomethacin treatment	2.5 ± 0.2^∗^	2.2 ± 1.0^∗^

*COX-2 ^-/-^ cells*		
Control (basal COX-1 activity)	8.9 ± 1.8	15.3 ± 2.8
Indomethacin treatment	5.3 ± 1.1^∗^	2.7 ± 0.6^∗^

*Note:*^∗^ indicates significant difference in the amount of PGE_2_ (mean ± SEM) spontaneously released by cells treated with indomethacin (5 μg/ml) in relation to the respective untreated control cells as determined by Student’s *t-*test, *n* = 3 (*P* < 0.05).

## Supplementary material

Supplementary Figure S1 Hierarchical clustering of gene expression in WT, COX-1^-/-^, and COX-2^-/-^ cells

Supplementary Figure S2 Hierarchical clustering of gene expression in WT, WT + IL-1β, COX-1^-/-^, and COX-2^-/-^ cells

Supplementary Figure S3 **Gene expression array of WT cells in the presence and absence of IL-1β** Six genes that were known to be upregulated by IL-1β were selected [21-25]. The basal expression of each gene in WT was represented as 1.0 and the corresponding ratio in IL-1β stimulated WT cells is presented. Expression of four housekeeping genes, including *β-actin*, *GAPDH*, *RPS13*, and *RPL30*, was also shown (average baseline fold change: 1.0 ± 0.2).

Supplementary Figure S4 **An integrated plan for omics analysis for eicosanoid metabolism** The biosynthesis of prostanoids and leukotrienes is summarized [37]. AA is a common intermediate for the COX and 5-LOX pathway, which is generated under the action of cPLA_2_. The COX-1 and COX-2 pathway generates several types of prostanoids. The stable end-products of eicosanoids were shown in boxes. cPLA_2_, cytosolic phospholipases A_2_; AA, arachidonic acid; LT, leukotriene; LOX, lipoxygenase; PG, prostaglandin; GST, glutathione *S*-transferase; TX, thromboxanes.

Supplementary Table S1 List of IL-1β-modulated transcripts in fibroblast cells

Supplementary Table S2 Differential expression of transcripts in WT, COX-1^-/-^, COX-2^-/-^, and WT+IL-1β- cells
