# Supplementary figures and images for: Genomic, Lipidomic and Metabolomic Analysis of Cyclooxygenase-null Cells: Eicosanoid Storm, Cross Talk, and Compensation by COX-1
Source: Genomics Proteomics Bioinformatics. 2016 Mar 21;14(2):81–93. doi: 10.1016/j.gpb.2014.09.005 (PMC4880957; doi:10.1016/j.gpb.2014.09.005)

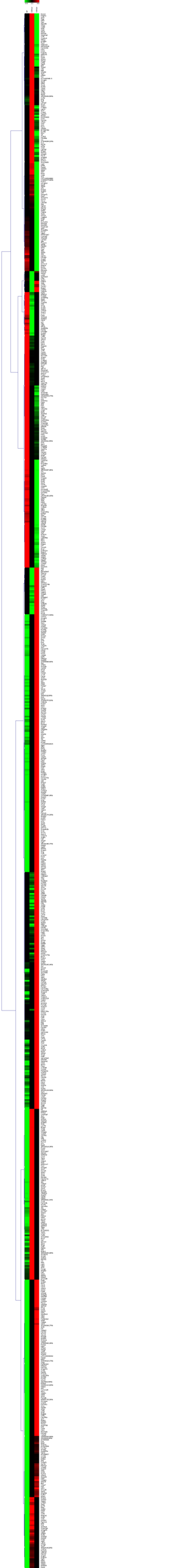

Supplement: Supplementary Figure S1 — Hierarchical clustering of gene expression in WT, COX-1-/-, and COX-2-/- cells [file mmc1.pdf]

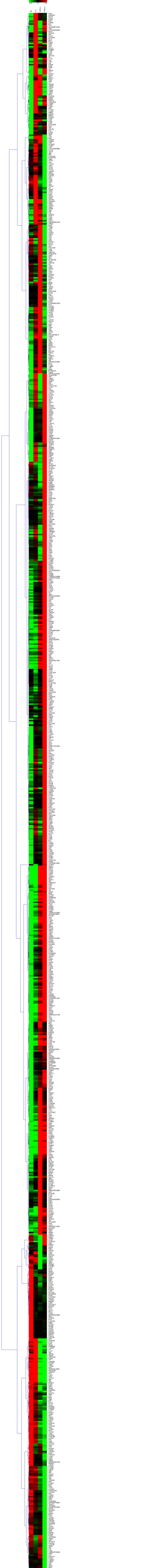

Supplement: Supplementary Figure S2 — Hierarchical clustering of gene expression in WT, WT + IL-1β, COX-1-/-, and COX-2-/- cells [file mmc2.pdf]

## Slide 1
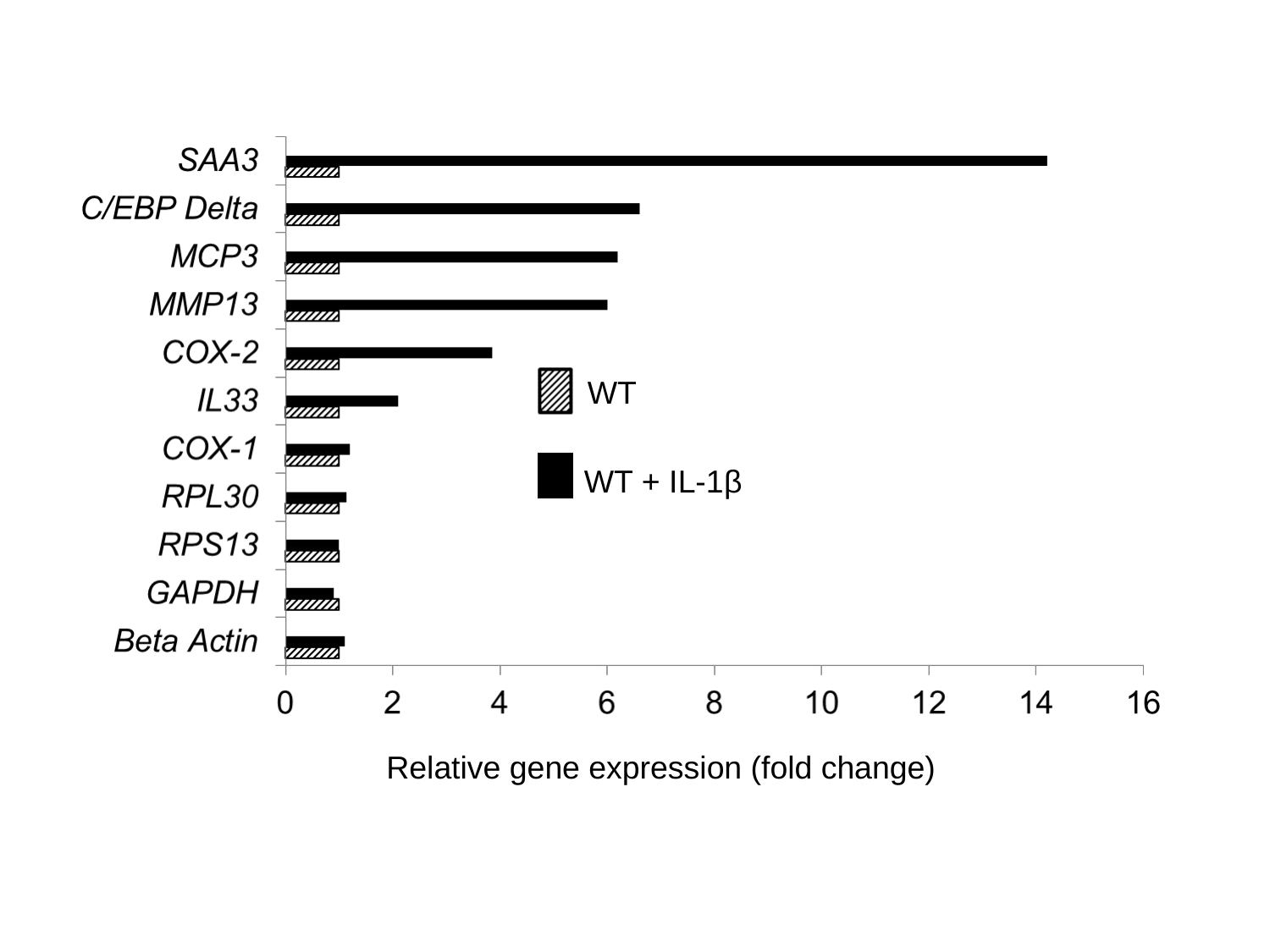

WT
WT + IL-1β
Relative gene expression (fold change)

Supplement: Supplementary Figure S3 — Gene expression array of WT cells in the presence and absence of IL-1β Six genes that were known to be upregulated by IL-1β were selected [21-25]. The basal expression of each gene in WT was represented as 1.0 and the corresponding ratio in IL-1β stimulated WT cells is presented. Expression of four housekeeping genes, including β-actin, GAPDH, RPS13, and RPL30, was also shown (average baseline fold change: 1.0 ± 0.2). [file mmc3.ppt]
