# Supplementary material for: Genomic, Lipidomic and Metabolomic Analysis of Cyclooxygenase-null Cells: Eicosanoid Storm, Cross Talk, and Compensation by COX-1
Source: Genomics Proteomics Bioinformatics. 2016 Mar 21;14(2):81–93. doi: 10.1016/j.gpb.2014.09.005 (PMC4880957; doi:10.1016/j.gpb.2014.09.005)
Supplement: Supplementary Figure S4 — An integrated plan for omics analysis for eicosanoid metabolism The biosynthesis of prostanoids and leukotrienes is summarized [37]. AA is a common intermediate for the COX and 5-LOX pathway, which is generated under the action of cPLA2. The COX-1 and COX-2 pathway generates several types of prostanoids. The stable end-products of eicosanoids were shown in boxes. cPLA2, cytosolic phospholipases A2; AA, arachidonic acid; LT, leukotriene; LOX, lipoxygenase; PG, prostaglandin; GST, glutathione S-transferase; TX, thromboxanes. [file mmc4.pptx]

## Slide 1
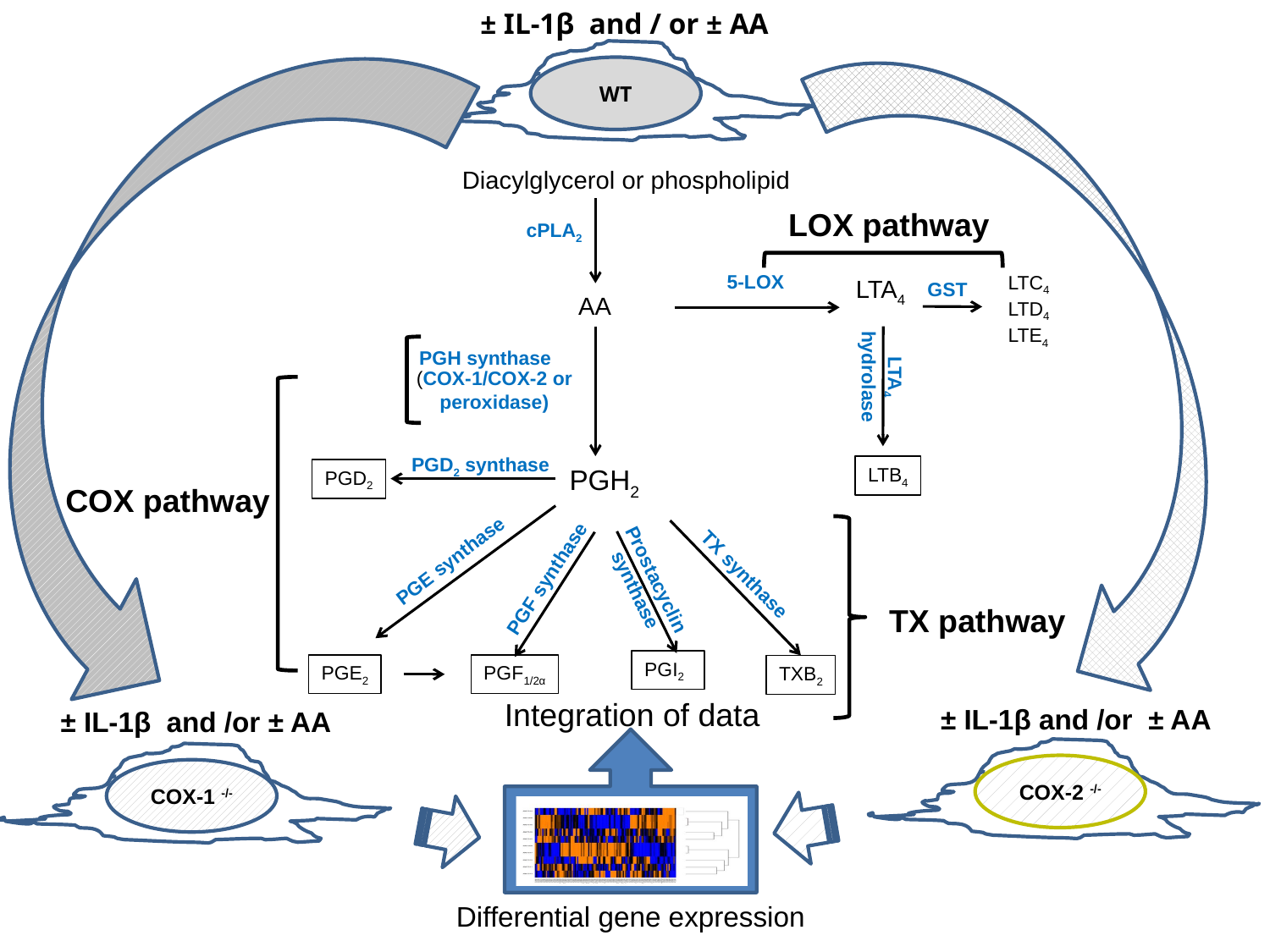

± IL-1β and / or ± AA
WT
Diacylglycerol or phospholipid
cPLA2
5-LOX
LTC4
LTD4
LTE4
LTA4
GST
AA
PGH synthase
LTA4 hydrolase
(COX-1/COX-2 or peroxidase)
PGD2 synthase
PGH2
LTB4
PGD2
PGE synthase
Prostacyclin
synthase
TX synthase
PGF synthase
PGE2
PGF1/2α
TXB2
LOX pathway
COX pathway
TX pathway
PGI2
Integration of data
± IL-1β and /or ± AA
± IL-1β and /or ± AA
COX-2 -/-
COX-1 -/-
Differential gene expression
