# Supplementary material for: Genomic, Lipidomic and Metabolomic Analysis of Cyclooxygenase-null Cells: Eicosanoid Storm, Cross Talk, and Compensation by COX-1
Source: Genomics Proteomics Bioinformatics. 2016 Mar 21;14(2):81–93. doi: 10.1016/j.gpb.2014.09.005 (PMC4880957; doi:10.1016/j.gpb.2014.09.005)
Supplement: Supplementary Table S2 — Differential expression of transcripts in WT, COX-1-/-, COX-2-/-, and WT+IL-1β- cells [file mmc6.docx]

**Table S2 Differential expression of transcripts in COX-1^-/-^, COX-2^-/-^, and WT+IL-1β cells**

| **No.** | **Gene name** | **Gene symbol** | **Fold change in relative to WT** | | |
| --- | --- | --- | --- | --- | --- |
|  |  |  | **WT+IL-1β** | **COX-1 ^-/-^** | **COX-2 ^-/-^** |
| 1 | Glutathione S-transferase, theta 1 | *Gstt1* | 1.26 | 0.91 | 13.47 |
| 2 | Annexin A8 | *Anxa8* | 1.80 | 0.69 | 6.26 |
| 3 | Peroxiredoxin 6 | *Prdx6* | 1.41 | 1.88 | 5.67 |
| 4 | NAD(P)H dehydrogenase, quinone 1 | *Nqo1* | 1.00 | 1.02 | 5.60 |
| 5 | Glutathione S-transferase alpha 4 | *Gsta4* | 1.05 | 1.37 | 5.56 |
| 6 | Prostaglandin-endoperoxide synthase 1 | *Ptgs1* | 1.03 | 0.56 | 4.30 |
| 7 | Inositol-3-phosphate synthase 1 | *Isyna1* | 1.44 | 1.43 | 3.51 |
| 8 | Glutathione S-transferase kappa 1 | *Gstk1* | 0.96 | 0.74 | 3.39 |
| 9 | Acyl-CoA synthetase long-chain family | *Acsl4* | 2.70 | 3.90 | 3.23 |
| 10 | Microsomal glutathione S-transferase 1 | *Mgst1* | 3.15 | 1.74 | 2.65 |
| 11 | Prostaglandin I2 (prostacyclin) synthase | *Ptgis* | 1.16 | 2.00 | 2.52 |
| 12 | Phospholipase A2, group VII | *Pla2g7* | 1.01 | 1.26 | 2.37 |
| 13 | Phosphate cytidylyltransferase 2 | *Pcyt2* | 1.06 | 1.45 | 2.33 |
| 14 | Microsomal glutathione S-transferase 2 | *Mgst2* | 1.36 | 1.51 | 2.32 |
| 15 | Aryl hydrocarbon receptor nuclear | *Ahr* | 1.55 | 1.33 | 2.25 |
| 16 | Nuclear factor of kappa (zeta) | *Nfkbiz* | 3.49 | 2.30 | 2.09 |
| 17 | Phospholipase A2, group IVA (cytosolic) | *Pla2g4a* | 1.65 | 1.18 | 2.07 |
| 18 | Phospholipid scramblase 2 | *Plscr2* | 1.51 | 0.78 | 2.05 |
| 19 | Sterol-C5-desaturase-like | *Sc5d* | 2.27 | 1.87 | 1.97 |
| 20 | Calcium-independent phospholipase A2 | *Pla2g6* | 1.47 | 1.19 | 1.74 |
| 21 | ELOVL family member 6, | *Elovl6* | 1.64 | 1.60 | 1.74 |
| 22 | Peroxisomal membrane protein PEX13 | *Pex13* | 1.52 | 1.83 | 1.74 |
| 23 | Phospholipid scramblase 1 i | *Plscr1* | 1.61 | 1.14 | 1.66 |
| 24 | Extracellular superoxide dismutase | *Sod3* | 1.42 | 0.60 | 1.64 |
| 25 | Superoxide dismutase 2, mitochondrial, | *Sod2* | 5.46 | 1.39 | 1.52 |
| 26 | Peroxiredoxin-5, mitochondrial | *Prdx5* | 1.16 | 1.11 | 1.49 |
| 27 | Fatty acid desaturase 1 | *Fads1* | 1.02 | 1.37 | 1.38 |
| 28 | Superoxide dismutase 1 | *Sod1* | 0.96 | 1.04 | 1.35 |
| 29 | Prostaglandin E receptor 4 (EP4) | *Ptger4* | 0.94 | 0.82 | 1.31 |
| 30 | Gamma-glutamyltransferase 1 | *Ggt1* | 1.12 | 1.20 | 1.31 |
| 31 | Prostaglandin reductase 1 | *Ptgr1* | 1.13 | 0.64 | 1.31 |
| 32 | Lipocalin-2 | *Lcn2* | 22.00 | 1.34 | 1.25 |
| 33 | Prostaglandin-endoperoxide synthase 2 | *Ptgs2* | 3.47 | 0.79 | 1.25 |
| 34 | Prostaglandin E synthase 2 | *Ptges2* | 1.17 | 1.21 | 1.23 |
| 35 | Microsomal glutathione S-transferase 3 | *Mgst3* | 0.83 | 0.89 | 1.22 |
| 36 | Prostaglandin F receptor (FP) | *Ptgfr* | 1.08 | 1.35 | 1.11 |
| 37 | 60S ribosomal protein L30 | *Rpl30* | 1.06 | 1.08 | 1.10 |
| 38 | G protein-coupled receptor 44 | *Gpr44* | 1.07 | 1.04 | 1.09 |
| 39 | Peroxisome proliferator-activated R γ | *Pparg* | 1.04 | 0.78 | 1.06 |
| 40 | Peroxisomal acyl-coenzyme A oxidase 1 | *Acox1* | 1.28 | 1.24 | 1.06 |
| 41 | Peroxisome proliferator-activated R d | *Ppard* | 0.98 | 1.04 | 1.05 |
| 42 | Leukotriene A4 hydrolase, | *Lta4h* | 0.77 | 1.17 | 1.05 |
| 43 | Non-specific lipid-transfer protein | *Scp2* | 1.15 | 1.39 | 1.04 |
| 44 | Leukotriene C4 synthase | *Ltc4s* | 1.03 | 1.02 | 1.03 |
| 45 | Leukotriene B4 receptor 1 | *Ltb4r1* | 1.18 | 1.01 | 1.02 |
| 46 | Thromboxane receptor | *Tbxa2r* | 1.04 | 1.00 | 1.01 |
| 47 | Formyl peptide receptor 1 | *Fpr1* | 1.00 | 1.02 | 1.01 |
| 48 | Phosphoglycerate dehydrogenase | *Phgdh* | 1.00 | 1.01 | 1.00 |
| 49 | 40S ribosomal protein S13 | *Rps13* | 1.02 | 0.98 | 1.00 |
| 50 | Thromboxane A synthase 1 | *Tbxas1* | 1.07 | 0.99 | 0.99 |
| 51 | Prostaglandin E receptor 1 | *Ptger1* | 0.97 | 1.02 | 0.99 |
| 52 | Prostaglandin E synthase 3 (cytosolic) | *Ptges3* | 0.89 | 0.98 | 0.98 |
| 53 | Carbonyl reductase 1, | *Cbr1* | 0.88 | 0.80 | 0.98 |
| 54 | Peroxisome proliferator-activated R | *Ppara* | 0.96 | 0.98 | 0.98 |
| 55 | Phosphatidylserine synthase 2 | *Ptdss2* | 1.10 | 1.24 | 0.97 |
| 56 | Arachidonate lipoxygenase 3 | *Aloxe3* | 0.95 | 0.96 | 0.93 |
| 57 | Prostaglandin D2 receptor (DP) | *Ptgdr* | 1.03 | 0.77 | 0.91 |
| 58 | Epoxide hydrolase 2, cytoplasmic | *Ephx2* | 0.77 | 0.80 | 0.89 |
| 59 | Hydroxysteroid (17-beta) dehydrogenase 4 | *Hsd17b4* | 1.13 | 1.15 | 0.80 |
| 60 | Formyl peptide receptor 2 | *Fpr2* | 0.74 | 0.77 | 0.79 |
| 61 | Gamma-glutamyltransferase 5 | *Ggt5* | 0.74 | 0.76 | 0.76 |
| 62 | Fatty acid synthase | *Fasn* | 0.87 | 0.68 | 0.74 |
| 63 | Dipeptidase 1 | *Dpep1* | 0.85 | 0.87 | 0.73 |
| 64 | Prostacyclin receptor | *Ptgir* | 0.96 | 0.78 | 0.69 |
| 65 | Prostaglandin E2 receptor | *Ptger2* | 0.65 | 0.60 | 0.67 |
| 66 | Beta-actin | *Actb* | 0.72 | 0.74 | 0.67 |
| 67 | Prostaglandin E receptor 3, | *Ptger3* | 0.64 | 0.66 | 0.66 |
| 68 | Eenoyl-CoA, hydratase | *Ehhadh* | 0.51 | 0.48 | 0.52 |

*Note:* The list shows transcripts with expression increased by 1.75 fold or decreased by 0.5 fold in COX-1^-/-^, COX-2^-/-^, or WT+IL-1β cells as compared to WT cells as ratios of IL-1β/WT, COX-1^-/-^/WT, and COX-2^-/-^/WT. The values in the WT cells were normalized as described in Materials and methods.
